# Lepromatous leprosy and perianal tuberculosis: a case report and literature review

**DOI:** 10.1186/1678-9199-20-38

**Published:** 2014-08-27

**Authors:** Maria Rita Parise-Fortes, Joel Carlos Lastória, Silvio Alencar Marques, Maria Stella Ayres Putinatti, Hamilton Ometto Stolf, Mariângela Ester Alencar Marques, Vidal Haddad

**Affiliations:** 1 Department of Dermatology, Botucatu Medical School, São Paulo State University (UNESP – Univ Estadual Paulista), Botucatu São Paulo State, Brasil; 2 Department of Pathology, Botucatu Medical School, São Paulo State University (UNESP – Univ Estadual Paulista), Botucatu, São Paulo State, Brazil; 3 Departamento de Dermatologia e Radioterapia, Faculdade de Medicina de Botucatu, UNESP, Distrito de Rubião Junior, Botucatu, SP CEP 18618-970, Brasil

**Keywords:** Mycobacteria, Leprosy, Tuberculosis, Erythema nodosum leprosum

## Abstract

Leprosy is a chronic infectious disease caused by *Mycobacterium leprae*, a microorganism that usually affects skin and nerves. Although it is usually well-controlled by multidrug therapy (MDT), the disease may be aggravated by acute inflammatory reaction episodes that cause permanent tissue damage particularly to peripheral nerves. Tuberculosis is predominantly a disease of the lungs; however, it may spread to other organs and cause an extrapulmonary infection. Both mycobacterial infections are endemic in developing countries including Brazil, and cases of coinfection have been reported in the last decade. Nevertheless, simultaneous occurrence of perianal cutaneous tuberculosis and erythema nodosum leprosum is very rare, even in countries where both mycobacterial infections are endemic.

## Background

*Mycobacterium leprae*, the causative agent of leprosy that affects the skin and peripheral nerves, shows tropism for macrophages and Schwann cells. The disease presents a wide clinical spectrum that is closely related to the patient specific immune response. The benign type, referred as tuberculoid or paucibacillary, is characterized by a Th1 immune response and production of type 1 cytokines including IFN-γ, IL-2, IL-12, IL-15 and TNF-α, which are typical of strong cell-mediated immunity. In lepromatous or multibacillary leprosy, the main characteristics comprise high levels of Th2-type cytokines such as IL-4, IL-5 and IL-10, high bacillary loads in skin lesions and reduced specific cellular immunity [1,2].

Tuberculosis is caused by *Mycobacterium tuberculosis* and is predominantly a disease of the lungs, with pulmonary tuberculosis accounting for 70% of cases [3]. *M. tuberculosis* can disseminate to other organs, including lymph nodes, bones, meninges and other extrapulmonary locations [3]. There are 9 million cases of active tuberculosis being reported annually and one third of the world’s population is supposed to be infected with *Mycobacterium tuberculosis*, although asymptomatically. Of these latent individuals, only 5-10% will develop active tuberculosis in their lifetime [3-5]. Similarly, in the natural history of leprosy, less than 5% of the people exposed to *M. leprae* will develop clinical disease [6,7].

Infections with intracellular pathogens such as *M. leprae* and *M. tuberculosis,* in most cases, are controlled by the cell-mediated immune response, based on CD4+ Th1 cells [8]. Leprosy is a more prevalent cause of cutaneous infections than tuberculosis and, even in endemic countries, the coinfection is uncommon in both diseases. We report in this paper a case of perianal tuberculosis in a patient whose lepromatous leprosy status was diagnosed as a type 2 leprosy reaction.

## Case report

**Figure 1:**
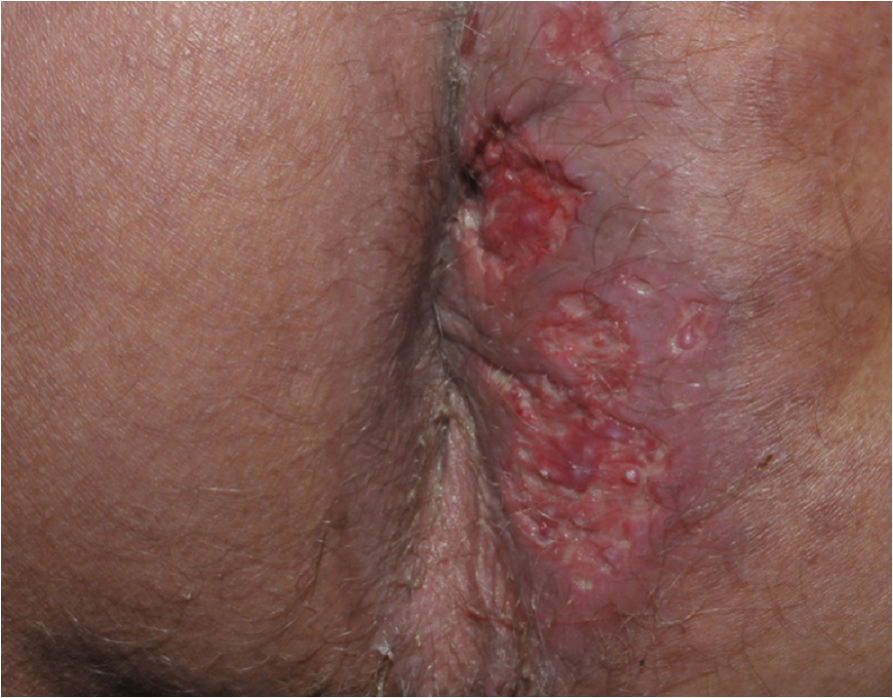
Cutaneous tuberculosis: fagedenic perianal ulcer.

**Figure 2:**
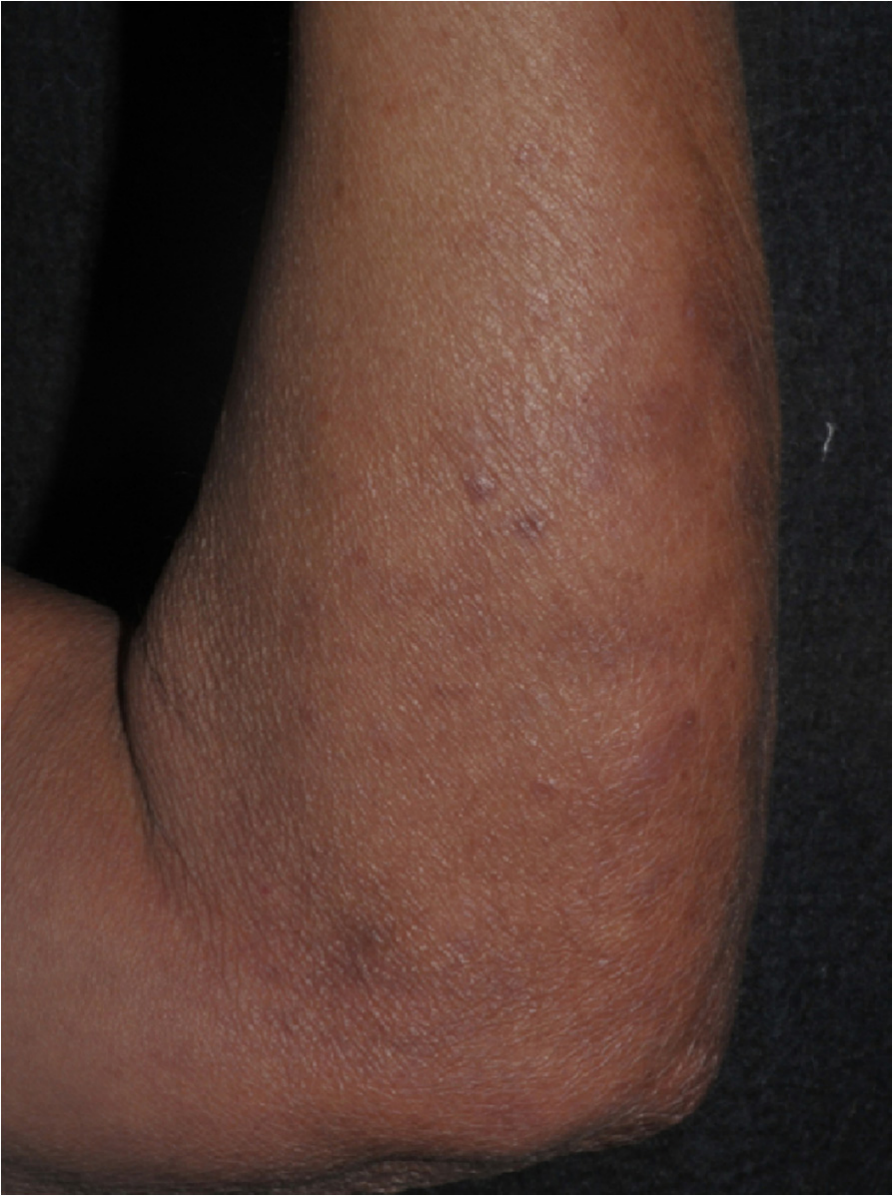
Lepromatous leprosy: erythematous papules and nodules on the upper limbs.

**Figure 3:**
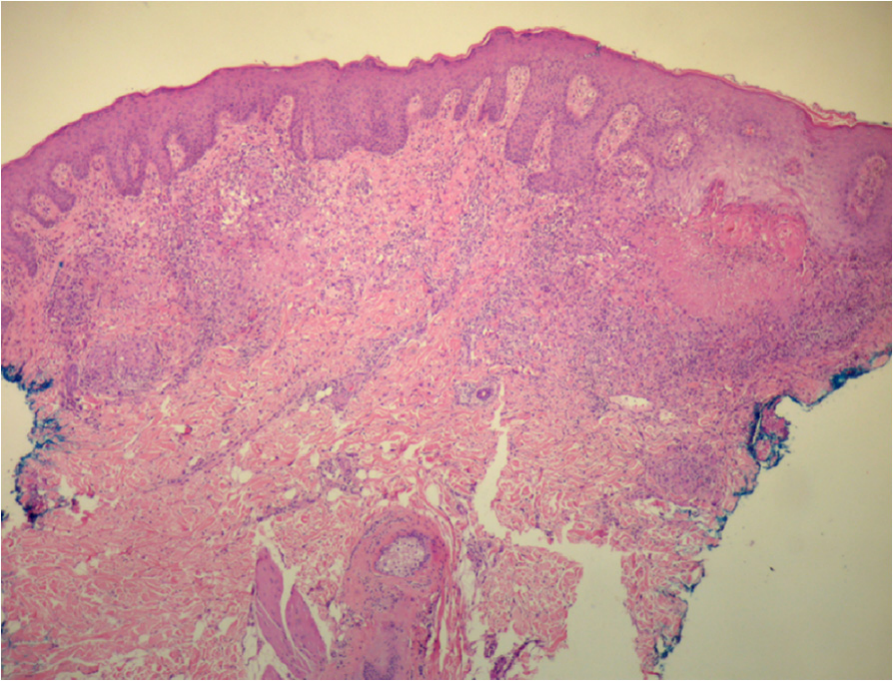
Histopathological exam of the perianal ulcer showing a dense histiocytic infiltrate and central caseous necrosis.

**Figure 4:**
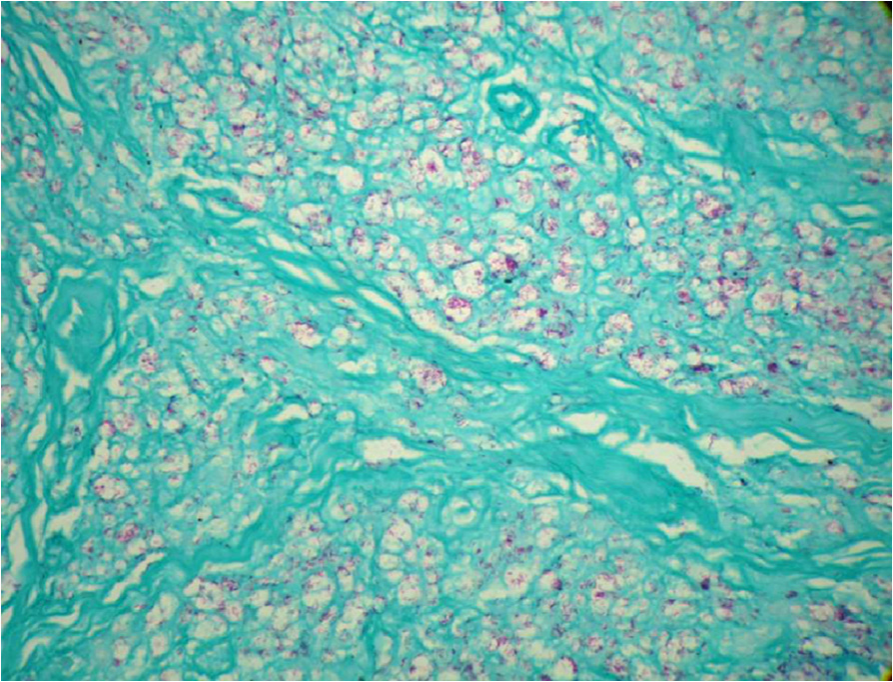
**Skin nodule consisting of foamy macrophages with globi of bacilli.** Fite-Faraco staining revealed the presence of lepromatous leprosy acid-fast bacilli.

A 59-year-old male patient was admitted to our university hospital (Botucatu, SP, Brazil) complaining of a perianal suppurative ulcer developed two months before the first medical consultation. He was under therapy with prednisone 40 mg/day for six months due to a presumed diagnosis of a drug reaction. On physical examination, a clean 10-cm diameter phagedenic perianal ulcer (Figure 1) and two longitudinal suppurative ulcers on the inguino-crural region were observed. During investigation, and soon after reducing the corticosteroid dose, disseminated erythematous papules and nodules were observed in the upper limbs (Figure 2).Histopathology of the perianal ulcer revealed a dense histiocytic dermal infiltrate and a granulomatous inflammatory response with central caseous necrosis (Figure 3). Fite-Faraco staining showed a few acid-fast bacilli and the hypothesis of cutaneous tuberculosis was raised. The histopathology of upper limbs papular and nodular lesions showed foamy macrophages with globi of bacilli, suggesting lepromatous leprosy (Figure 4). The perianal and inguinal ulcers were considered uncommon manifestations of type 2 leprosy developed when corticosteroid was gradually diminished. The patient was treated as for multibacillary leprosy with multidrug therapy and thalidomide.

**Figure 5:**
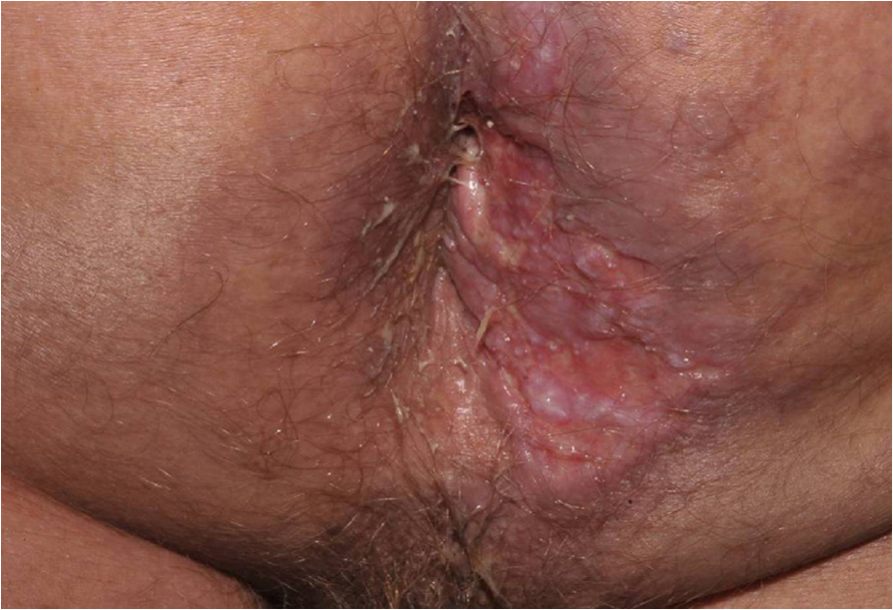
Cutaneous tuberculosis: phagedenic perianal ulcer showing worsening of the perianal ulcer after treatment for leprosy lesion, for two months.

**Figure 6:**
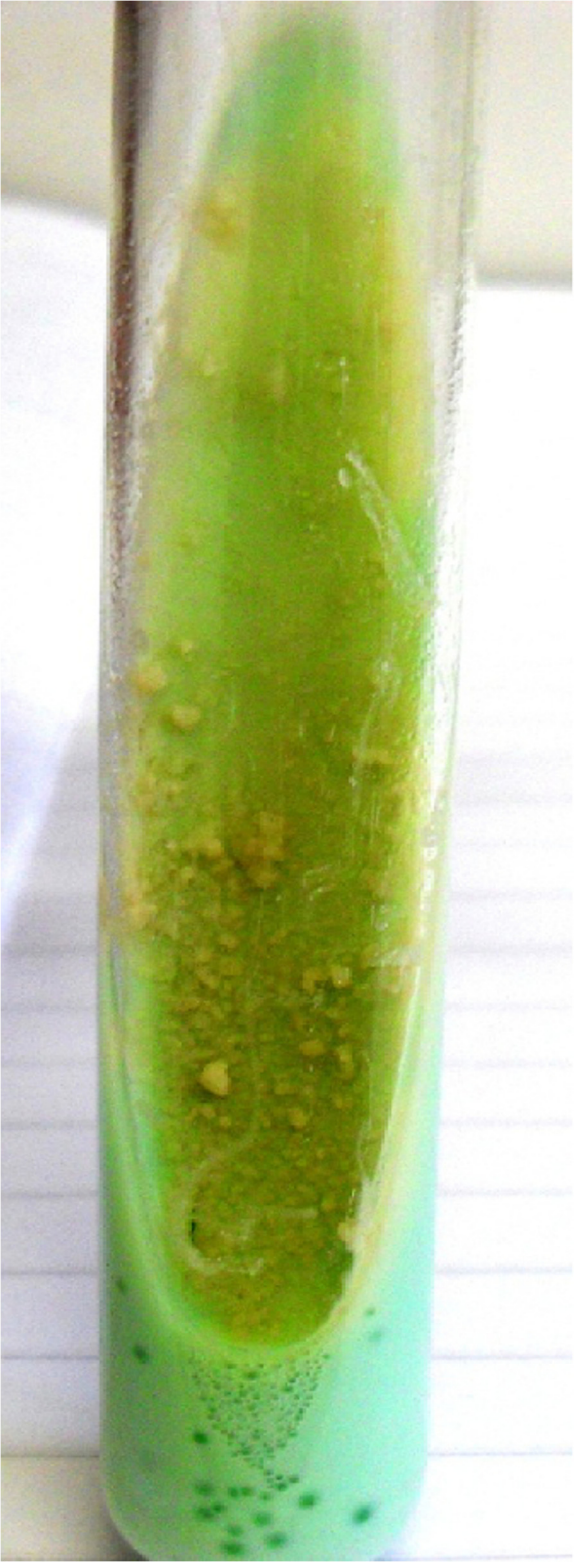
***M. tuberculosis, *** **positive culture on Löwenstein-Jensen medium at 37°C.**

**Figure 7:**
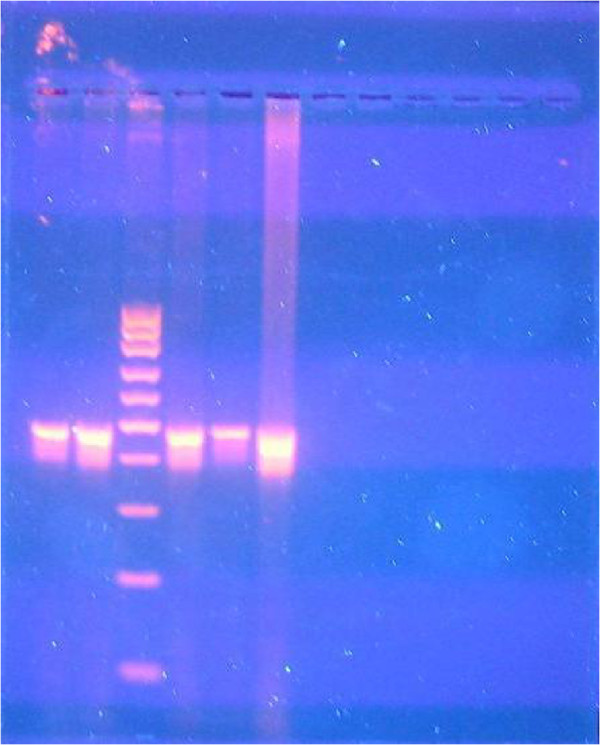
***M. leprae*, positive polymerase chain reaction DNA analysis.**

**Figure 8:**
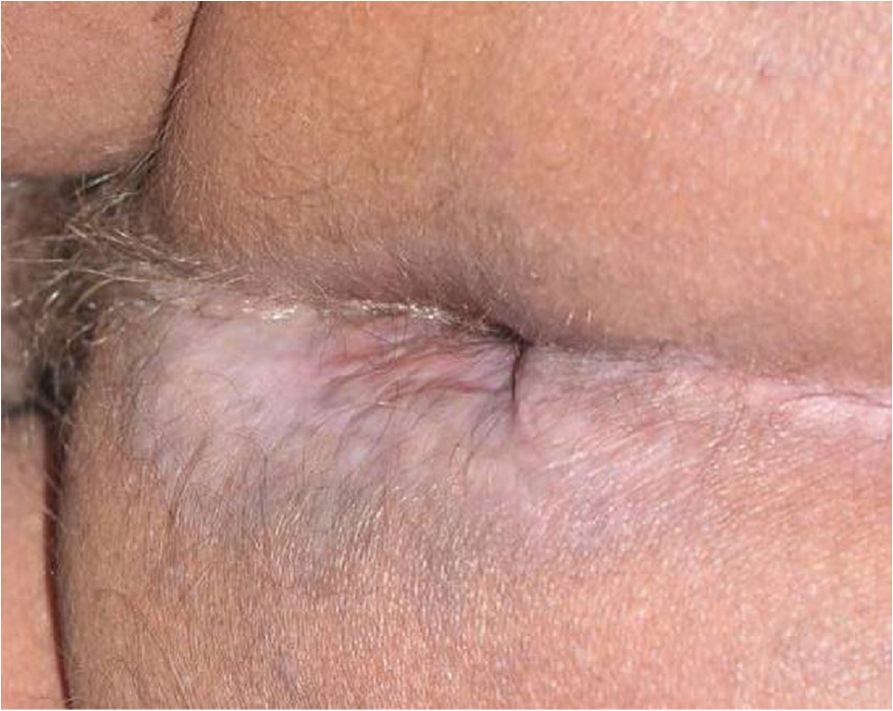
Perianal ulcer completely healed with antituberculous treatment for two months.

Four weeks later, the patient had fever, weight loss, asthenia and worsening of the perianal ulcer (Figure 5), but without erythema nodosum leprosum. With this clinical picture, new investigation was developed. The tuberculin skin test was positive (20 × 20 mm) and culture of *M. tuberculosis* – using a biopsy sample from the perianal ulcer – on Löwenstein-Jensen medium was positive (Figure 6). Polymerase chain reaction (PCR) and DNA analysis from upper limb skin samples were positive for *M. leprae* (Figure 7). With the new information the patient was finally diagnosed as presenting simultaneously lepromatous leprosy – type 2 reaction – and cutaneous tuberculosis expressed by the perianal ulcer. Screening for HIV, HBV or HCV infection as well as lung, renal or colon compromising were negative. Hence, the patient was treated with isoniazid, ethambutol and rifampin for nine months with complete healing of the perianal and inguinal lesions after two months (Figure 8). After the tuberculosis treatment, the patient was submitted to additional multidrug therapy for 12 months with dapsone, rifampin and clofazimine with complete cure of leprosy.

## Discussion

Tuberculosis is one of the most important health concerns in the world, which causes relevant levels of morbidity and mortality, particularly in many developing countries, in spite of this being the era of human immunodeficiency virus infection [9]. The number of tuberculosis cases in industrialized and developing countries has increased in recent years, followed by increasing multidrug resistance [10].

Multidrug resistance and HIV infection are predisposing factors for the disease, since the immune response of the patient plays a significant role on clinical manifestation of tuberculosis [10-12]. Extrapulmonary tuberculosis accounts for 13% of all cases [4]. Nevertheless, cutaneous tuberculosis is uncommon and is reported to occur in less than 1.5% of the cases of extrapulmonary tuberculosis [13].

The present study reports a clinical case of perianal tuberculosis and lepromatous leprosy with type 2 reaction coinfection without any detected active pulmonary or gastrointestinal infection. The association between lepromatous leprosy and perianal tuberculosis with erythema nodosum leprosum demonstrates an extremely uncommon occurrence.

Perianal tuberculosis may manifest in the following forms: ulcer, fistula, abscess, lupoid infiltration or miliary. The most common type is the ulcerative lesion, which is likely to have well-defined boundaries and is characterized by mucopurulent discharge [14]. The lesion observed in the present report was ulcerative with many bacilli, signaling an immunosuppressive status probably related to corticosteroid therapy.

Perianal tuberculosis has rarely been reported, even in countries where the disease is endemic, and the diagnosis is impaired due to the lack of a pulmonary focus. The possibility of an association with intestinal tuberculosis should be further investigated [15,16].

Coinfection with pulmonary tuberculosis and leprosy type 1 reaction has been reported and the leprosy type 1 reaction was observed during the treatment of pulmonary tuberculosis [17]. According to Trindade *et al*. [17], a review of patients between 2004 and 2011 showed that only two patients with leprosy were diagnosed as coinfected with pulmonary tuberculosis. The occurrence of both tuberculosis and leprosy in the same individual is not an unusual clinical condition, but it is scarcely reported in the literature, even in countries where both diseases are endemic [18].

Delobel *et al.*[19] reported a triple association of American cutaneous leishmaniasis, lepromatous leprosy and pulmonary tuberculosis. The authors suggested that the unresponsiveness of patient’s T cells to IL-12 *in vitro,* stimulated by either *L. guyanensis, M. bovis* BCG or *M. leprae* antigens, could be the evidence of patient’s T cells failure to produce an appropriate Th cell response. This was responsible for the triple reported coinfection. There are other reports in the literature of coinfection of leprosy and pulmonary tuberculosis as well as leprosy and disseminated tuberculosis in HIV-infected patients [20-24].

Leprosy is a disease of poverty. Its diagnosis remains based on clinical signs and symptoms. Delayed diagnosis associated with nerve impairment and physical deformities is still common in several endemic areas throughout the globe. Improving the medical attention and infrastructure and promoting sanitary education will provide earlier diagnosis and specific treatment, which are crucial to help interrupt the *M. leprae* transmission chain [25,26]. Both leprosy and tuberculosis pose significant health risks, making it important for physicians to diagnose accurately and provide appropriate treatment for patients [27].

CD4+ T cells, as well as the cytokines IL-12, IFN-γ and TNF-α, are critical in the control of *M. tuberculosis* and *M. leprae* infections, but the host factors that determine why some individuals are protected from infection while others develop the disease are still unclear [28]. Hence, the reason why *M. tuberculosis* and *M. leprae* are able to evade host immune surveillance and persist inside the macrophages remains to be understood. These pathogens inhibit phagosome-lysosome fusion and stop phagosome maturation at an early stage, thus allowing escape from microbicidal peptides [29].

Genetic factors of the host and of the pathogen itself may be associated with an increased risk for patients to develop active tuberculosis and leprosy [28]. The immunologic deficits observed in leprosy are specific for *M. leprae*. Immunosupression has been observed to render individuals susceptible to *M. leprae* and *M. tuberculosis* in cases of transplantation, cancer chemotherapy and in treatment with antitumor necrosis factor agents [30-32]. However, the coinfection with lepromatous leprosy and tuberculosis probably depends on multiple factors including low socioeconomic status, poor nutrition, chemotherapy-induced immunosuppression and deficient host immune response [27].

The present report shows a rare case which was probably eased by the immunosuppressive effect of corticosteroid therapy for six months. Cutaneous tuberculosis remains to be one of the most difficult conditions to diagnose in developing countries due to the lack of resources and the necessary observation of clinical and histopathologic findings [11,33,34]. To the best of our knowledge, this is the first case of lepromatous leprosy associated with erythema nodosum leprosum and perianal tuberculosis fully documented.

## Conclusion

In the present study, we report an association of lepromatous leprosy with type 2 reaction and perianal ulcerative tuberculosis without previous or active pulmonary infection. Simultaneous occurrence of cutaneous tuberculosis and lepromatous leprosy is extremely infrequent, even in countries where both mycobacterial infections are endemic.

### Ethics committee approval

The case report was submitted for analysis and approved by the Research Ethics Committee of the Botucatu Medical School (CEP), in accordance with resolution 466/2012.

## Consent

Written informed consent was obtained from the patient for publication of this case report and the figures.

## Competing interests

The authors declare that they have no competing interests.

## Authors’ contributions

All authors contributed to the design of the study and manuscript preparation. All authors have read and approved the final manuscript.
